# Comment on “A quantitative definition of hypervalency” by M. C. Durrant, *Chem. Sci.*, 2015, **6**, 6614

**DOI:** 10.1039/c5sc04866d

**Published:** 2016-02-25

**Authors:** Richard D. Harcourt, Thomas M. Klapötke

**Affiliations:** a School of Chemistry , The University of Melbourne , Victoria 3010 , Australia . Email: r.harcourt@unimelb.edu.au ; Fax: +61 3 93475180; b Department of Chemistry , Ludwig-Maximilian University of Munich , Butenandstr. 5-13(D) , D-81377 Munich , Germany

## Abstract

Consideration is given to (electronically) hypervalent increased-valence structures, which possess 2c–1e bonds, fractional 2c–2e bonds, and usually normal 2c–2e bonds. For singlet-spin electron-rich systems, increased-valence structures, with Heitler–London 2c–2e bond wavefunctions, are equivalent to resonance between non-hypervalent Kekulé and Dewar (or singlet diradical) type Lewis structures. Dewar structures are not considered in the *Chem. Sci.* 2015, **6**, 6614 Edge article on hypervalency. Using one-electron delocalizations from lone-pair atomic orbitals into separate bonding molecular orbitals, increased-valence structures for PCl_5_, O_3_, SO_4_^2–^, NO_3_^–^, N_2_O_4_ and S_N_2 reactions are derived from the Edge-article's Kekulé-type Lewis structures, and compared with the Edge article's hypervalent structures with 2c–2e bonds. It is also shown that Durrant's method to determine the *γ* parameter for **XAY**-type systems that possess a symmetrical 3c–4e bonding unit is related to the **A**-atom charge density.

## Abstract 2

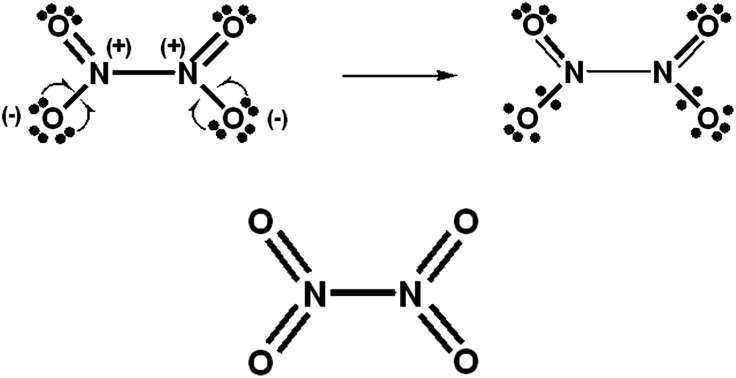
Consideration is given to (electronically) hypervalent increased-valence structures, which possess 2c–1e bonds, fractional 2c–2e bonds, and usually normal 2c–2e bonds.



## Introduction

Durrant^1^ has provided a quantitative definition of hypervalency *via* reference to atomic charges, which were obtained either from experimental or from theoretical electron densities using Bader's quantum theory of atoms in molecules (QTAIM) methodology.^3^ Without calculation it was not usually possible to conclude whether a given molecule was hypervalent. For example, the hypercoordinate molecules CLi_6_ and SiH_6_^2–^ were calculated^1^ to be hypervalent and non-hypervalent, respectively.^4^

For this comment on hypervalence (primarily for electron-rich molecules), we use and discuss types of hypervalent VB structures that were not considered in ref. 1, and which, since 1968,^8^,^9^ have been designated as “increased-valence” structures without expansion of valence shells. They involve 2-centre, 1-electron (2c–1e) bonds and fractional 2c–2e bonds (with bond-numbers less than unity, and represented by thin bond lines),^6^,^8^,^9^ and usually non-fractional 2c–2e bonds.

**Scheme 1:**
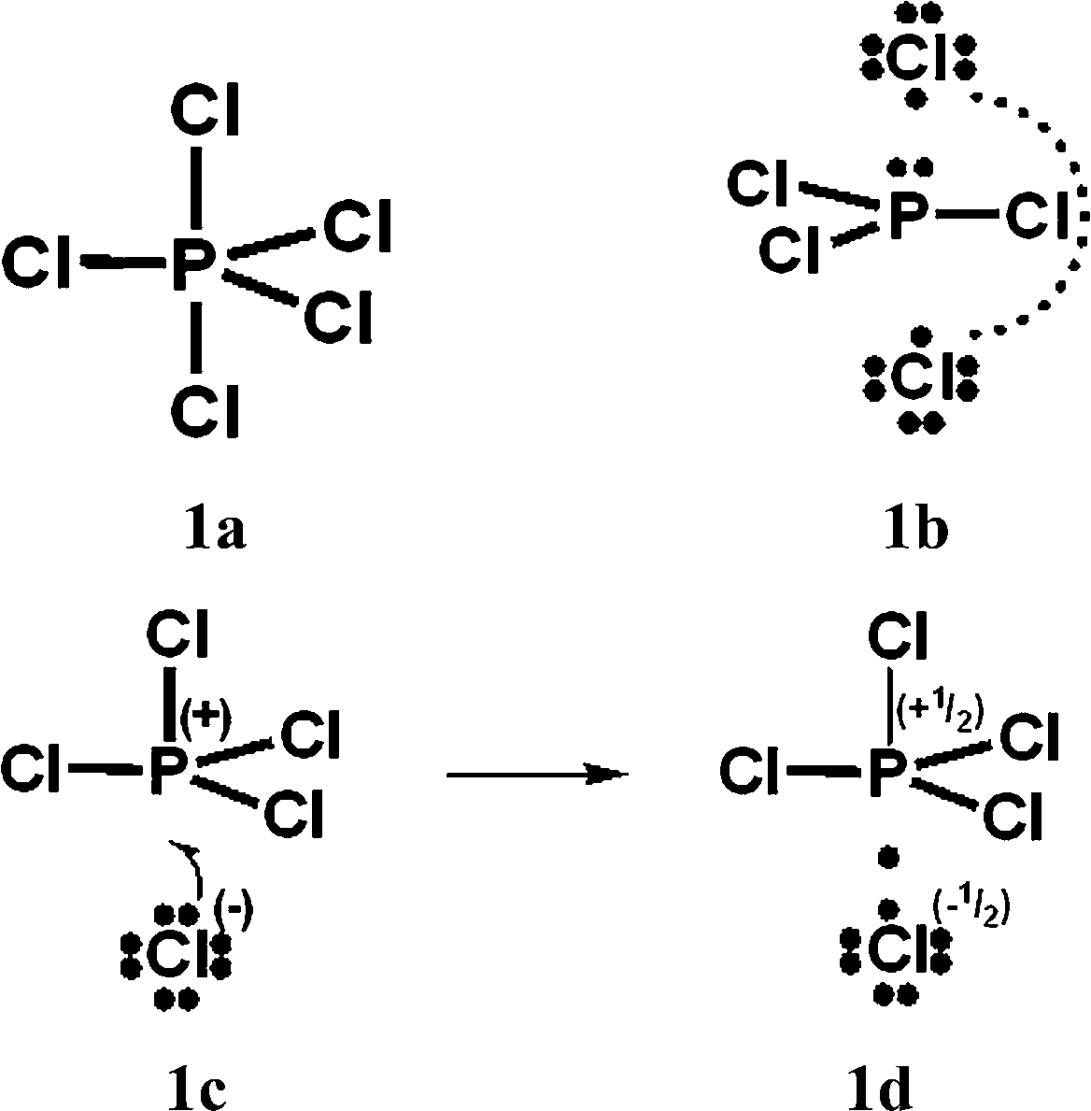
Hypercoordinate PCl_5_ valence-bond (VB) structures. Here and in Schemes 2–6, (non-variationally best) atomic formal charges for increased-valence structures are assigned on the assumption that bonding electrons are shared equally by pairs of adjacent atoms,^9^ and mirror-image structures are not displayed. Of course the extent of delocalization differs for non-equivalent 3c, 4e bonding units.

**Scheme 2:**
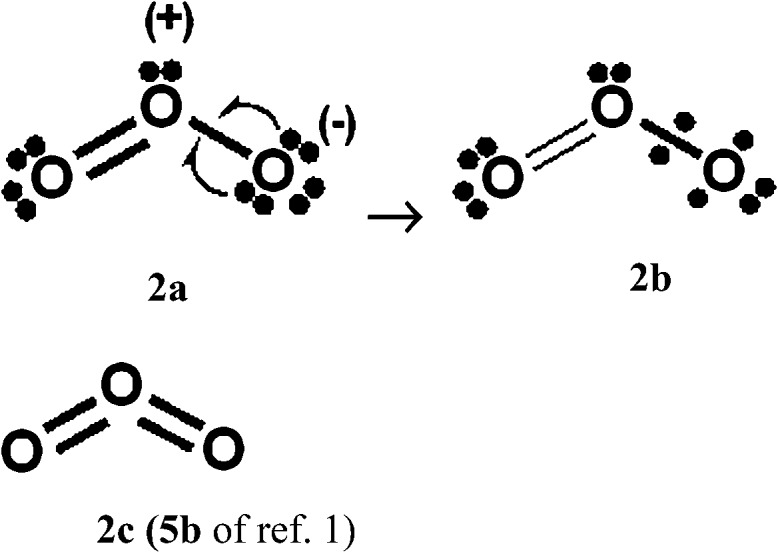
O_3_ VB structures.

In Schemes 1, 2 and 4–6, increased-valence structures for PCl_5_, O_3_, SO_4_^2–^, NO_3_^–^ and N_2_O_4_ are generated from Lewis octet VB structures and compared with the non-octet hypervalent VB structures displayed in ref. 1, namely structures **1a**, **2c**, **4c**, **5c** and **6c** here. Brief consideration is also given to increased-valence structures for S_N_2 reactions.

For illustrative purposes, in the Appendix we show that Durrant's method to determine the *γ* parameter of ref. 1 for **XAY**-type systems with a symmetrical 3c–4e bonding unit is related to the **A**-atom charge density obtained from a 3c–4e molecular orbital (MO) configuration. Consideration is also given to the **A**-atom valence.

### Increased-valence structures and hypervalence

For PCl_5_, O_3_, SO_4_^2–^, NO_3_^–^ and N_2_O_4_, Durrant^1^ has displayed two types of Lewis VB structures that involve non-fractional (2c–2e) bonds between pairs of adjacent atoms, namely familiar, non-hypervalent octet Kekulé type structures **1c**, **2a**, **3a**, **4a**, **5a** and **6a**, and the hypervalent structures **1a**, **2c**, **4c**, **5c**, and **6c** that violate the octet rule. For electron-rich hypercoordinate molecules, such as PCl_5_ (*cf.* Scheme 1 of ref. 1 and Scheme 1 here) and for the electron-rich non-hypercoordinate molecules and ions O_3_, SO_4_^2–^, NO_3_^–^ and N_2_O_4_, 2c–2e bonds are the only types of bonds that are present in both types of Lewis VB structures.

Dewar/singlet diradical types of Lewis VB structures, such as **1b** and **3b–3d**, were not considered in ref. 1. In ref. 6, it is indicated that hypervalency for electron-rich systems arises when Dewar/type structures participate in resonance with the Kekulé type structures.

When relevant atomic orbitals (AOs) overlap, Lewis-type VB structures for electron-rich species can be stabilized *via* 1-electron delocalizations from separate lone-pair AOs into 2-centre bonding MOs or bond orbitals (BOs), as is shown, for example, in structures **1c** for PCl_5_ in Scheme 1, and **2a**, **4a**, **5a** and **6a** for O_3_, SO_4_^2–^, NO_3_^–^ and N_2_O_4_ in Schemes 2 and 4–6. The resulting VB structures, **1d**, **2b**, **4b**, **5b** and **6b** possess (thin-bond line) fractional 2c–2e bonds and 2c–1e bonds, as well as normal 2c–2e bonds.

By inspection, one can see that more electrons participate in bonding in VB structures **1d**, **2b**, **4b**, **5b** and **6b** than does occur in the Lewis Kekulé structures **1c**, **2a**, **4a**, **5a** and **6a**. Therefore **1d**, **2b**, **4b**, **5b** and **6b** are examples of “increased-valence^”^ structures^6^,^8^,^9^ without expansion of the valence shell. Because relative to the octet Lewis structures, increased-valence structures involve additional electrons in both nearest-neighbour and non-neighbour bonding, increased-valence structures are hypervalent relative to the Lewis structures.^6^,^8^,^9^

### Some properties of increased-valence structures

**Scheme 3:**
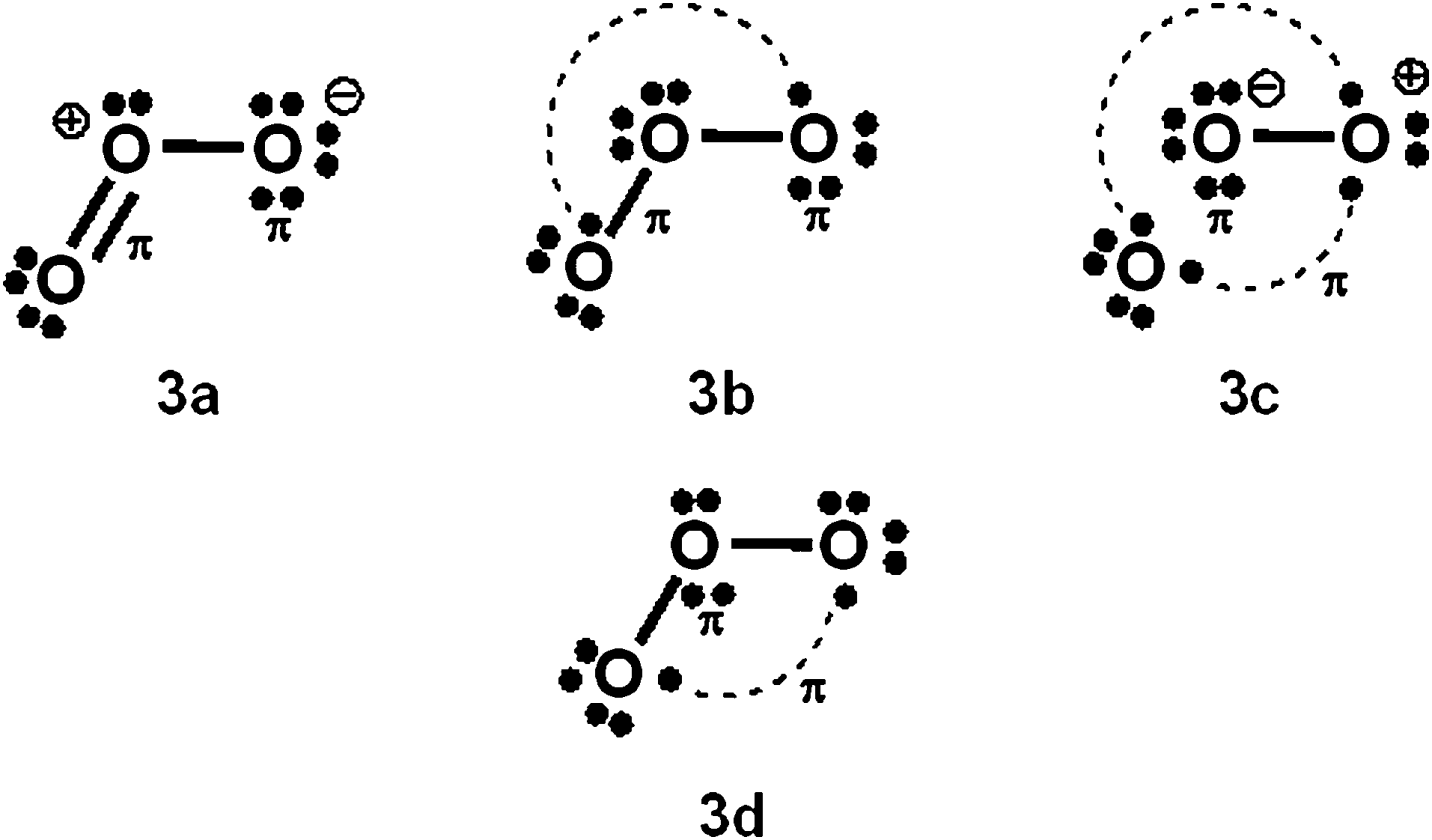
Component Kekulé and Dewar/singlet diradical Lewis structures for increased-valence structure **2b** of Scheme 2.

With Heitler–London AO type wavefunctions for 2c–2e bonds – for example *a*(1)*b*(2) + *b*(1)*a*(2) for the 2c–2e **A–B** bond – increased-valence structures for electron-rich molecules summarize resonance between two types of Lewis structures, namely the familiar, standard/Kekulé type Lewis octet structures, and “long-bond”/formal bond/singlet diradical/Dewar type Lewis (octet) structures. As indicated already, the latter type of Lewis VB structure is not considered in ref. 1. For the O_3_ increased-valence structure **2b**, these two types of Lewis octet structures (namely structure **3a** and structures **3b–3d**) are displayed in Scheme 3. None of them is hypervalent, but resonance between them generates hypervalence for the resulting increased-valence structure.

The PCl_5_ and O_3_ Dewar structures **1b**, **3b** and **3d** do not carry atomic formal charges, and **1b** and **3d** in particular were not considered by Durrant.

For an increased-valence structure that does not involve a valence shell expansion to provide an additional AO for bonding, none of the component Lewis structures violates the octet rule, but resonance between them to generate the increased-valence structure leads to (an apparent) violation of the octet rule.^6^,^8^,^9^

### Increased-valence structures for SO_4_^2–^, NO_3_^–^ and N_2_O_4_

**Scheme 4:**
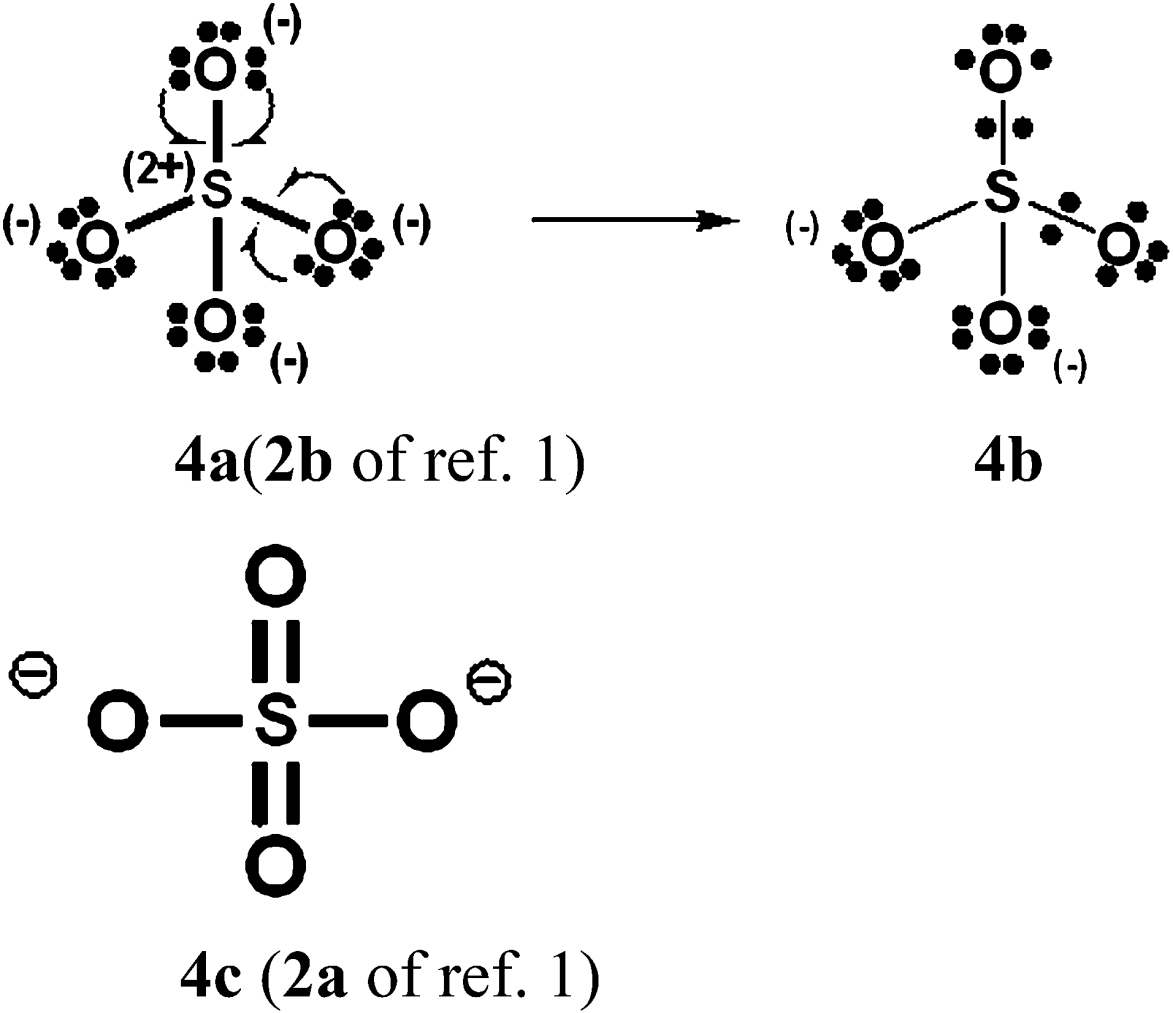
SO_4_^2–^. As well as structure **4b**, another type of increased-valence structure can be constructed with four fractional 2c–2e bonds + four 2c–1e bonds and oxygen atom formal charges of –1/2.

**Scheme 5:**
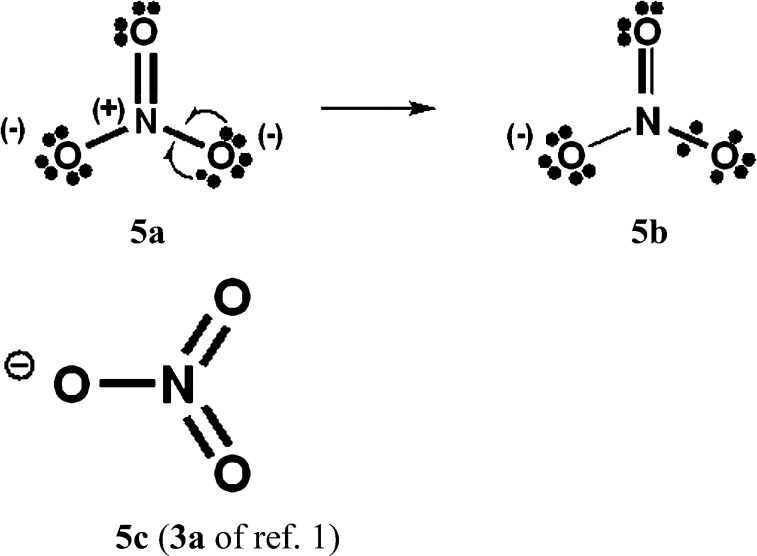
NO_3_^–^. As well as **5b**, another type of increased-valence structure can be constructed, with two fractional 2c–2e bonds and two oxygen atoms with formal charges of –1/2.

**Scheme 6:**
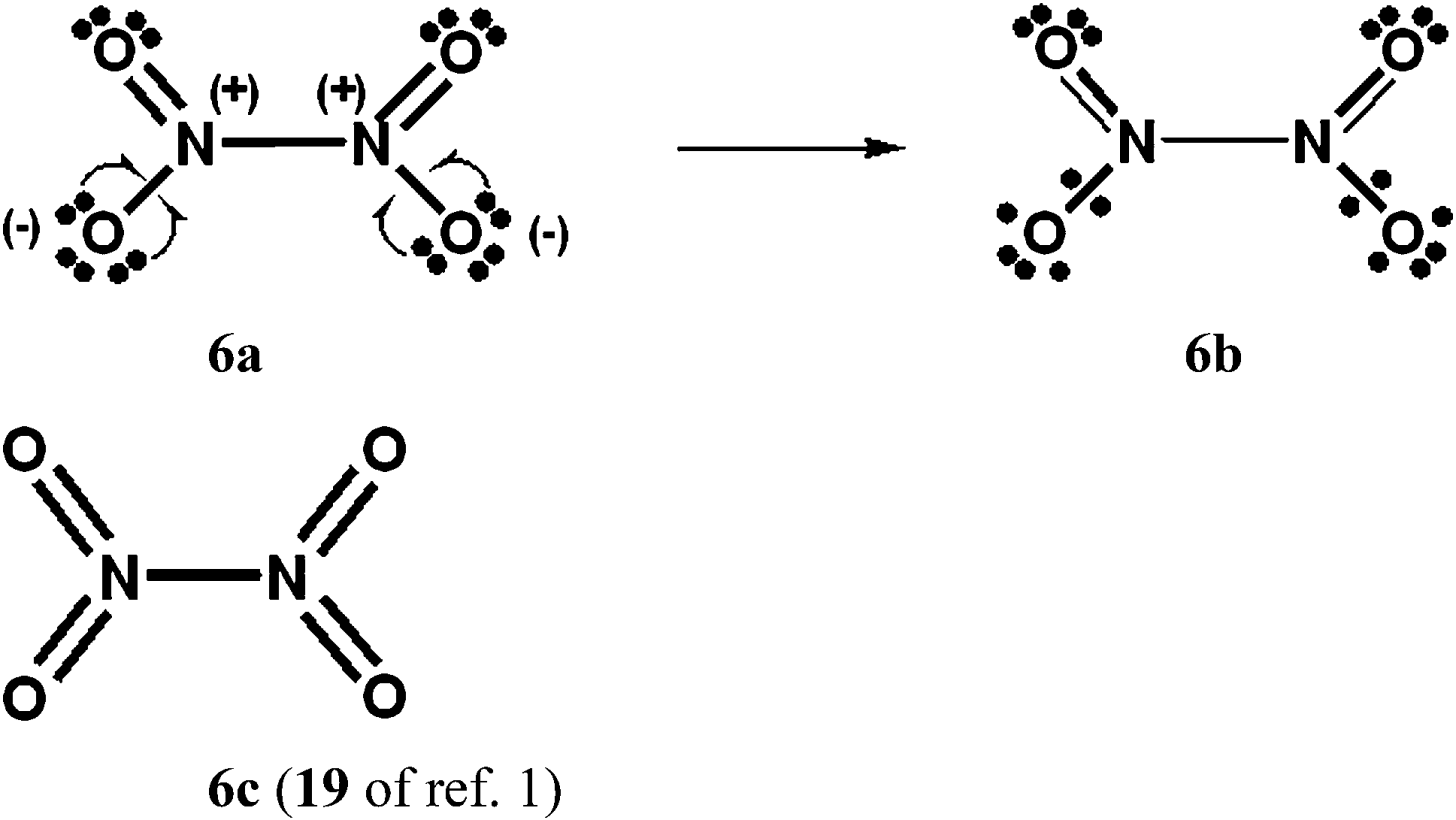
N_2_O_4_ with *D*_2h_ symmetry. The 16 component Kekulé and Dewar Lewis structures are displayed in ref. 9*a* and *b*, as well as an increased-valence structure for an ONONO_2_ isomer. There are two “*cis*” type structures for each of **6a** and **6b**, and two “*trans*” type structures.

In Schemes 4–6, we have generated increased-valence structures for SO_4_^2–^, NO_3_^–^ and N_2_O_4_ from familiar octet Kekulé type Lewis structures *via* 2c–1e delocalizations of oxygen O^–^ electrons. In these figures, the hypervalent VB structures with only 2c–2e bonds displayed in ref. 1 are also displayed. We suggest that the increased-valence structures with 1-electron bonds and fractional 2c–2e bonds provide better insight into the possible origin of some molecular properties than do the hypervalent VB structures of ref. 1. For example:

(a) The fractional N–N 2c–2e bond in the increased-valence structure **6b** for N_2_O_4_ is in accord with the presence of a long, weak N–N single bond.^9^

(b) The results of VB calculations for O_3_ and related 1,3-dipolar systems from numerous laboratories – see for example ref. 7*d* and *e* and 10 and references therein – indicate that their ground-states possess substantial singlet-diradical character. It arises primarily from the contribution of the Lewis structure **3d** of Scheme 3 to the ground-state Lewis structure resonance scheme. In contrast to structure **2c** (*i.e.* structure **5b** of ref. 1), increased-valence structure **2b** of Scheme 2 reflects the diradical character.

### S_N_2 reactions

With Coulson–Fischer^11^ type BOs (*a* + *k*_1_*b*) and (*b* + *k*_2_*a*) replacing the *a* and *b* AOs of the Heitler–London wavefunction for a 2c–2e bond, the course of an S_N_2 reaction, **X^–^** + **AY** → **XA** + **Y^–^** has been formulated^12^ as
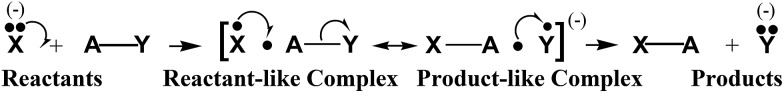


For it, the increased-valence structures for the **(XAY)^–^** reactant-like and product-like complexes, each with an additional bonding electron relative to the **AY** reactant and **XA** product, are both hypercoordinate and hypervalent relative to the VB structures for the latter species.

### Further comments and conclusion

When 2-centre Coulson–Fisher type BOs, such as the *a* + *k*_1_*b* and *b* + *k*_2_*a* above, are used to accommodate the electrons that form the fractional 2c, 2e bonds in the increased-valence structures, allowance can be made for polarization of these bonds.

In ref. 13, the wavefunctions for 3c–4e VB structures of the types **X**—**A**—**Y** (as would occur in the Durrant structures **2c**, **5c** and **6c** for O_3_, NO_3_^–^ and N_2_O_4_ if expansion of the valence shell does not occur) and •**X** • **A** • **Y**•, and the Rundle-Hach^14^–Pimentel^15^ 3c–4e MO configuration have been shown to be special cases of wavefunctions for resonance between the increased-valence structures •**X** • **A**—**Y** and **X**—**A** • **Y**•, with 2-centre Coulson–Fischer orbitals, and one variational parameter.^16^

Increased-valence structures can also be constructed for: (a) systems that involve 3c–3e bonding units,^17^ with non-reactant and non-product ionic structures replacing the singlet-diradical structures of electron-rich systems, and (b) diatomic molecules.^18^

Regardless of the method used to construct the wave-functions for increased-valence structures, because they involve the participation of more electrons in bonding than do the Kekulé-type Lewis structures from which they are derived, increased-valence structures are hypervalent relative to these Lewis structures. As indicated above, for electron-rich systems, this is due to the inclusion of singlet diradical structures in the Lewis structure resonance scheme. Also, increased-valence structures involve at least one Pauling three-electron bond as a diatomic component.^9^

## Appendix: valence electron equivalent parameter *γ*(**A**) and atomic valencies

In ref. 1, the valence electron equivalent parameter *γ*(**A**) for atom **A** is used to determine whether a molecule exhibits hypervalence.^19^ Here for the linear, symmetrical, triatomic systems of Table 1, each with one 3c–4e bonding unit, we shall show that the Durrant method^1^ to construct *γ*(**A**) is equivalent to a method that uses a 3c–4e MO configuration with AO overlap integrals omitted.

**Table 1:** *γ*(**A**) and **A-**atom valencies

Molecule	**A**-atom charge^1^	*k* ^2^	*γ*(**A**)	**A**-Atom valence
F_3_^–^	–0.056	2.13_7_	8.11	1.35
ClF_2_^–^	+0.291	1.09_8_	7.42	1.11
Cl_3_^–^	–0.019	2.07_7_	8.04	1.34
BrCl_2_^–^	+0.108	1.16_0_	7.78	1.27
ICl_2_^–^	+0.263	1.16_7_	7.47	1.14
XeF_2_	+1.230	1.25_2_	7.54	1.17
KrF_2_	+1.003	1.98_8_	7.99	1.06
XeCl_2_	+0.763	3.24_2_	8.47	1.37

### MO wavefunction

As the wavefunction for the 3c–4e electrons, we shall use the Hach-Rundle–Pimentel MO configuration^14^,^15^ of eqn (1).

1 |*ψ*_1_^*α*^*ψ*_1_^*β*^*ψ*_2_^*α*^*ψ*_2_^*β*^| ∝ |(2*x* + *ka*)^*α*^(2*x* + *ka*)^*β*^(2*y* + *ka*)^*α*^(2*y* + *ka*)^*β*^|

The *x*, *a* and *y* are the overlapping AOs on the three atomic centres, and *ψ*_1_ = *x* + *ka* + *y* and *ψ*_2_ = *x* – *y* are the bonding and non-bonding MOs that can be constructed from them.^14^,^15^

The right-hand side of eqn (1) gives the valence-bond structure^21^ (**X**—**A**—**Y**)^*q*^, with fractional 2c–2e bonds that arise from double-occupation of two non-orthogonal BOs, and *q* = –1 or 0.

To determine the value for *k*, we equate the charge *Q*_**A**_ of ref. 1 for atom **A** to *X***_A_** – 2*k*^2^/(*k*^2^ + 2). The *X***_A_** is the core charge of atom **A** when the 3c–4e electrons are removed, and 2*k*^2^/(*k*^2^ + 2) = *P*_aa_ is the **A**-atom charge density that arises from the 3c–4e bonding. For the neutral species and anions of Table 1, *X***_A_** = 2 and 1, respectively.

### Durrant's method^1^ to construct *γ*(**A**)

To construct the *γ*(**A**) parameter for the systems considered in Table 1, initially we follow Durrant's methodology,^1^ as described in ref. 1 for CO, and for SCl_4_ in the ESI for ref. 1. We use the expanded valence-shell (hypervalent) covalent structure **X <svg xmlns="http://www.w3.org/2000/svg" version="1.0" width="16.000000pt" height="16.000000pt" viewBox="0 0 16.000000 16.000000" preserveAspectRatio="xMidYMid meet"><metadata> Created by potrace 1.16, written by Peter Selinger 2001-2019 </metadata><g transform="translate(1.000000,15.000000) scale(0.005147,-0.005147)" fill="currentColor" stroke="none"><path d="M0 1520 l0 -160 1360 0 1360 0 0 160 0 160 -1360 0 -1360 0 0 -160z"/></g></svg>** **A**^(*q*)^** <svg xmlns="http://www.w3.org/2000/svg" version="1.0" width="16.000000pt" height="16.000000pt" viewBox="0 0 16.000000 16.000000" preserveAspectRatio="xMidYMid meet"><metadata> Created by potrace 1.16, written by Peter Selinger 2001-2019 </metadata><g transform="translate(1.000000,15.000000) scale(0.005147,-0.005147)" fill="currentColor" stroke="none"><path d="M0 1520 l0 -160 1360 0 1360 0 0 160 0 160 -1360 0 -1360 0 0 -160z"/></g></svg>** **Y** (with two non-fractional 2c–2e bonds) and the ionic structure **X**^(–)^**A**^(*q*+2)^**Y**^(–)^ (with *q* = *X***_A_** – 2).

These structures are weighted according to the value of *q* = *X***_A_** – 2 so that the QTAIM charge *Q*_**A**_ of ref. 1 is reproduced *via*eqn (2)

2 *xq* + (1 – *x*)(*q* + 2) = *Q***_A_**

to give *x* = (*X***_A_** – *Q***_A_**)/2.

The *γ*(**A**) is then calculated from eqn (4)*via*eqn (3),

3 *γ*(**A**) = 10*x* + 6(1 – *x*)

4 = 6 + 2(*X***_A_** – *Q***_A_**)

in which 10 and 6 are the number of **A**-atom electrons associated with the covalent and ionic structures.

### MO method to construct *γ*(**A**)

Alternatively we can use the 3c–4e MO configuration of eqn (1), and a *k*-dependent probably density function (*P*(*k*)) for the 3c–4e bonding unit. If we choose *P*(*k*) = 4*k*^2^/(*k*^2^ + 2) ≡ 2*P*_aa_, with *Q***_A_** = *X***_A_** – 2*k*^2^/(*k*^2^ + 2), the resulting *γ*(**A**) parameter is given by eqn (6)*via*eqn (5).

5 *γ*(**A**) = 6 + 4*k*^2^/(*k*^2^ + 2)

6 = 6 + 2(*X***_A_** – *Q***_A_**)

Therefore eqn (3) is identical to eqn (5), thereby showing that the Durrant methodology to determine *γ*(**A**) for a symmetric 3c–4e bonding unit corresponds to using a contribution by the **A**-atom's charge density to a MO formulation of 3c–4e bonding.

Three of the calculated values for *γ*(**A**) reported in Table 1 are greater than 8, which indicates hypervalency^1^ for the associated species. However of course different numerical values and conclusions would be obtained with different types of 3c–4e wavefunctions and *P*(*k*) functions.

### **A**-Atom valence

In ref. 22, with AO overlap integrals omitted, it is deduced that the **A**-atom valence (*V***_A_**) for the MO configuration of eqn (1) is given by eqn (7),

7 *V***_A_** = *V*_*ax*_ + *V*_*ay*_ = 16*k*^2^/{(*k*^2^ + 4)(*k*^2^ + 2)}

with a maximum value of 1.3726 when *k*^2^ = 2√2.

In Table 1, the MO estimates of the **A** atom valence for each species exceeds unity, and therefore its **A** atom is hypervalent. However, regardless of the values of the **A**-atom valence and the *γ*(**A**), because of the presence of 2c–1e bonds in addition to the 2c–2e bonds, more electrons participate in nearest-neighbour and non-neighbour bonding^6^,^8^,^9^ for each of the increased-valence structures than does occur in any of their component Lewis structures. Therefore all increased-valence structures are electronically hypervalent.

## Note added in proof

For PCl_5_, an increased-valence analogue of structure **1a** is obtained *via* the delocalization of two electrons from the Cl^–^ of the Kekulé structure **1c** rather than one (as in structure **1c**), to give two 1-electron P–Cl bonds, a fractional equatorial 2c–2e P–Cl bond as well as the fractional axial 2c–2e P–Cl bond of structure **1d**.
